# Structural durability of early-generation Transcatheter aortic valve replacement valves compared with surgical aortic valve replacement valves in heart valve surgery: a systematic review and meta-analysis

**DOI:** 10.1186/s13019-020-01170-7

**Published:** 2020-06-08

**Authors:** Ashlynn Ler, Yeo Jie Ying, Faizus Sazzad, Andrew M. T. L. Choong, Theo Kofidis

**Affiliations:** 1 Department of Cardiac, Thoracic and Vascular Surgery, National University Heart Centre, 9th Floor, Tower Block, 1E Kent Ridge Road, Singapore, 119228 Singapore; 2 School of Medicine, National University of Ireland, Galway, Ireland; 3 School of Medicine, Queen’s University Belfast, Belfast, UK; 4 Department of Surgery, Yong Loo Lin School of Medicine, National University of Singapore, Kent Ridge Road, Singapore; 5 Cardiovascular Research Institute, MD6, 14 Medical Drive, National University of Singapore, Singapore, 117599 Singapore; 6 National University Health System (NUHS), 5 Lower Kent Ridge Road, Kent Ridge Road, 119228 Singapore

**Keywords:** SAVR valves, TAVR valves, Structural durability

## Abstract

**Background:**

The current treatment for aortic stenosis includes open surgical aortic valve replacement (SAVR) as well as endovascular transcatheter aortic valve replacement (TAVR). This study aims to compare the 1-year, 2–3 year and 5-year structural durability of TAVR valves with that of SAVR valves.

**Method:**

A systematic literature search was conducted in July 2019 on Medline (via PubMed), Embase and Cochrane electronic databases according to the PRISMA guidelines.

**Results:**

Thirteen randomized controlled trials were included. From the meta-analysis, we observed higher rates of 1-year (OR: 7.65, CI: 4.57 to 12.79, *p* < 0.00001), 2–3-year (OR: 13.49, CI: 5.66 to 32.16, *p* < 0.00001) and 5-year paravalvular regurgitation (OR: 14.51, CI: 4.47 to 47.09, *p* < 0.00001) associated with the TAVR valves than the SAVR valves. There were also higher rates of 1-year (OR: 5.00, CI: 3.27 to 7.67, *p* < 0.00001), 2–3-year (OR: 8.14, CI: 3.58 to 18.50, *p* < 0.00001) and 5-year moderate or severe aortic regurgitation (MD: 14.65, CI: 4.55 to 47.19, *p* < 0.00001), and higher rates of 1-year (OR: 3.55, CI: 1.86 to 6.77, *p* = 0.0001), 2–3-year (OR: 3.55, CI: 1.86 to 6.77, *p* = 0.0001) and 5-year reintervention (OR: 3.55, CI: 1.22 to 10.38, *p* = 0.02) in the TAVR valves as compared to SAVR valves.

**Conclusion:**

TAVR valves appear to be more susceptible to structural valve deterioration and thus potentially less structurally durable than SAVR valves, given that they may be associated with higher rates of moderate or severe aortic regurgitation, paravalvular regurgitation and reintervention in the 1-year-, 2–3 year, and 5-year period.

## Introduction

Aortic stenosis is currently the most common valvular disease in developed countries, with an overall prevalence of approximately 1–3% in European patients who are more than 70 years old [1]. The current methods for treatment for the disease include Surgical Aortic Valve Replacement (SAVR) and the less-invasive Transcatheter Aortic Valve Replacement (TAVR) technique. Traditionally, TAVR procedures were reserved for high-risk patients or patients with severe symptomatic aortic valve stenosis [2]. In recent years, the use of this technique has been extended to low-and intermediate-risk patients as well [3], with more studies evaluating the safety and efficacy of this minimally invasive procedure in these differing patient cohorts.

Both SAVR and TAVR use bioprosthetic valves, with the SAVR valve being a fixed stent with an estimated life span of 15 years and the TAVR valve being capable of expanding and collapsing [4]. However, TAVR being the newer procedure, with the first valve implanted in 2002 by Alan Cribier [5], and developments in the technique and valves having spanned only just under 20 years, the life span of the TAVR valve is still uncertain. Currently, studies that compare TAVR to SAVR valves report data of only up to 5 or 6 years, making an assessment of valve durability beyond that time frame difficult to determine. Additionally, while much has been done on determining the clinical outcomes of TAVR patients in single-arm studies, relatively fewer studies have reported data on the 5-year structural durability of the TAVR valves in comparison with SAVR valves. Hence, the present study aims to compare the 1-year, 2–3 year and 5-year structural durability of the early-generation TAVR valves as compared to SAVR valves.

## Methods

A systematic review was conducted according to the Preferred Reporting Items for Systematic Reviews and Meta-analyses for systematic review (PRISMA) standard [6]. We conducted electronic searches on Medline (via PubMed), Embase and Cochrane database records from the date of inception to 3 July 2019. On the PubMed database, a repetitive and exhaustive combination of the following search terms were used: “Transcatheter aortic valve replacement valve durability”, “Aortic valve replacement valve durability comparison”, “Surgical aortic valve replacement valve durability comparison”, “SAVR TAVR valve durability”, “Surgical aortic valve replacement versus transcatheter aortic valve replacement valve durability” and “Durability for aortic bioprosthesis for TAVR”.

### Inclusion criteria and exclusion criteria

Any randomized controlled trials that reported both SAVR and TAVR valve structural durability in patients. Animal studies, case reports, survey results, laboratory studies and any studies that were not written in the English language were excluded, as well as reports on haemodynamic simulations and studies that focused on the quality of life of SAVR and TAVR patients.

### Study selection

Three reviewers (A.L, Y.J.Y, F.S) screened and assessed the studies independently for inclusion. The articles were first screened by their titles and abstracts. The full-text review was performed on articles if the reviewer was unable to confirm the relevance of the study for inclusion.

### Quality of evidence and risk of bias assessment

As illustrated in chapter 11 of the Cochrane handbook of reviews [7], GRADEpro was used to evaluate the quality of evidence in the included studies (Table 1). Two reviewers (A.L, Y.J.Y) assessed the articles for their risk of bias and quality of evidence. Risk of bias of each study was assessed according to guidelines in chapter 8 of the Cochrane handbook of reviews [13] (Fig. E1) and risk of bias plots were generated using RevMan 5 [14] (Fig. E2).

**Table 1:** Quality of Evidence of Included Studies

Certainty assessment							№ of patients		Effect		Certainty	Importance
№ of studies	Study design	Risk of bias	Inconsistency	Indirectness	Imprecision	Other considerations	TAVR valve	SAVR valve	Relative (95% CI)	Absolute (95% CI)		
Transcatheter aortic-valve replacement with a self-expanding prosthesis^15^												
1	randomised trials	not serious	not serious	not serious	not serious	none	390/747 (52.2%)	357/747 (47.8%)	not estimable		⨁⨁⨁⨁ HIGH	CRITICAL
3-Year Outcomes in High-Risk Patients Who Underwent Surgical or Transcatheter Aortic Valve Replacement^16^												
1	randomised trials	not serious	not serious	not serious	not serious	none	391/750 (52.1%)	359/750 (47.9%)	not estimable		⨁⨁⨁⨁ HIGH	CRITICAL
Longitudinal Hemodynamics of Transcatheter and Surgical Aortic Valves in the PARTNER Trial^17^												
1	randomised trials	serious ^a,b^	not serious	not serious	not serious	none	2482/2795 (88.8%)	313/2795 (11.2%)	not estimable		⨁⨁⨁◯ MODERATE	CRITICAL
5-Year Outcomes of Self-Expanding Transcatheter Versus Surgical Aortic Valve Replacement in High-Risk Patients^18^												
1	randomised trials	not serious	not serious	not serious	not serious	none	390/744 (52.4%)	354/744 (47.6%)	not estimable		⨁⨁⨁⨁ HIGH	CRITICAL
Comparison of Transcatheter and Surgical Aortic Valve Replacement in Severe Aortic Stenosis: A Longitudinal Study of Echo Parameters in Cohort A of the PARTNER Trial^19^												
1	randomised trials	serious ^b^	not serious	not serious	not serious	none	348/699 (49.8%)	351/699 (50.2%)	not estimable		⨁⨁⨁◯ MODERATE	CRITICAL
Transcatheter or Surgical Aortic-Valve Replacement in Intermediate-Risk Patients^20^												
1	randomised trials	not serious	not serious	not serious	not serious	none	1101/2032 (54.2%)	1021/2032 (50.2%)	not estimable		⨁⨁⨁⨁ HIGH	CRITICAL
Self-Expanding Transcatheter Aortic Valve Replacement Versus Surgical Valve Replacement in Patients at High Risk for Surgery A Study of Echocardiographic Change and Risk Prediction^21^												
1	randomised trials	serious ^b^	not serious	not serious	not serious	none	389/795 (48.9%)	353/795 (44.4%)	not estimable		⨁⨁⨁◯ MODERATE	CRITICAL
5-year outcomes of transcatheter aortic valve replacement or surgical aortic valve replacement for high surgical risk patients with aortic stenosis (PARTNER 1): a randomised trial^22^												
1	randomised trials	serious ^a^	not serious	not serious	not serious	none	348/699 (49.8%)	351/699 (50.2%)	not estimable		⨁⨁⨁◯ MODERATE	CRITICAL
Surgical or Transcatheter Aortic-Valve Replacement in Intermediate-Risk Patients [9]												
1	randomised trials	not serious	not serious	not serious	not serious	none	864/1660 (52.0%)	796/1660 (48.0%)	not estimable		⨁⨁⨁⨁ HIGH	CRITICAL
Durability of Transcatheter and Surgical Bioprosthetic Aortic Valves in Patients at Lower Surgical Risk [10]												
1	randomised trials	not serious	not serious	not serious	not serious	none	139/274 (50.7%)	135/274 (49.3%)	not estimable		⨁⨁⨁⨁ HIGH	CRITICAL
Five-Year Clinical and Echocardiographic Outcomes From the NOTION Randomized Clinical Trial in Patients at Lower Surgical Risk [11]												
1	randomised trials	not serious	not serious	not serious	not serious	none	145/280 (51.8%)	135/280 (48.2%)	not estimable		⨁⨁⨁⨁HIGH	CRITICAL
Transcatheter Versus Surgical Aortic Valve Replacement in Patients With Severe Aortic Valve Stenosis: 1-Year Results From the All-Comers NOTION Randomized Clinical Trial [12]												
1	randomised trials	not serious	not serious	not serious	not serious	none	145/280 (51.8%)	135/280 (48.2%)	not estimable		⨁⨁⨁⨁ HIGH	CRITICAL
Transcatheter Aortic-Valve Replacement with a Self-Expanding Valve in Low-Risk Patients [8]												
1	randomised trials	not serious	not serious	not serious	not serious	none	725/1403 (51.7%)	678/1403 (48.3%)	not estimable		⨁⨁⨁⨁ HIGH	CRITICAL

*CI* Confidence interval

Explanations

^a^. Patients and their treating physicians were not masked to treatment allocation

^b^. Attrition bias due to amount, nature or handling of incomplete outcome data

### Data abstraction and outcomes of interest

Two authors (A.L, Y.J.Y) independently abstracted details of the study population. Data extracted included: Title, authors, year of publication, study type, number of patients, sex, age, body surface area, NHYA class III or IV, and histories of hypertension, peripheral vascular disease, pulmonary disease, coronary artery disease, diabetes mellitus, prior coronary artery bypass grafting, prior atrial fibrillation, prior myocardial infarction, pre-existing pacemaker and prior balloon valvuloplasty.

The primary outcome measures were 1-year, 2–3 year and 5-year moderate or severe aortic regurgitation, valve endocarditis and reintervention rate. The secondary outcome measures were all-cause mortality and specific mortality, which is defined as mortality due to specifically cardiovascular causes.

### Author-defined time frames

Following full-text review of the included studies, we observed the presence of heterogeneity in the length of follow-up period in each study. In order to resolve this, we defined the follow up time frames into three categories: Patient data reported within the first year, patient data reported in 2–3 years post valve implantation and patient data reported within a 5-year (or more) period post-operation.

### Author-defined aortic regurgitation

For studies that reported moderate and severe aortic regurgitation as separate values, we calculated ‘moderate and severe aortic regurgitation’ by adding together the combined incidence of moderate and severe aortic regurgitation.

### Statistical analyses

All forest plots were generated using RevMan 5 [14]. All meta-analyses were carried out using random-effects models to account for statistical variability across the studies. Where absolute numbers were not explicitly stated, the percentages reported were taken and multiplied to the total number of participants to obtain the number of events. These values were rounded up when the first decimal place was above 5 and rounded down when the first decimal place was less than 5. For all forest plots, we compared odds ratios (OR) and the confidence intervals (CI) of these odds ratios across the studies.

## Results

**Fig. 1:**
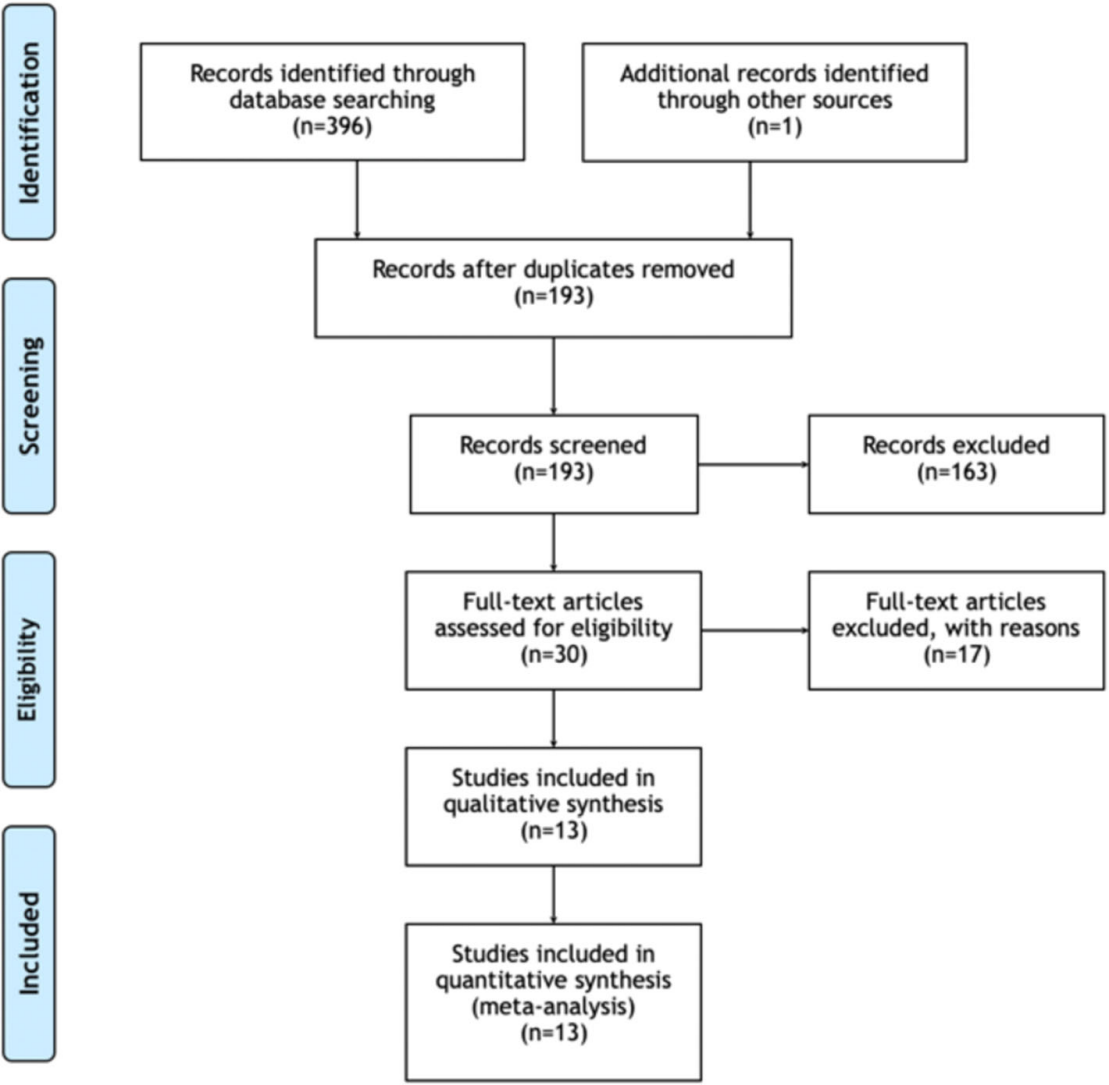
PRISMA chart illustrating our process of obtaining the 13 included articles. Our initial search produced 396 studies and 1 study was retrieved from alternative sources. Out of this initial pool of studies, 193 remained after duplicates were removed. With 163 irrelevant records excluded based on their titles and abstracts, we reviewed the full texts of 30 articles, of which 17 were excluded and 13 remained for inclusion in our study

The systematic search revealed a total of 396 papers. One paper was retrieved from alternative sources. After duplicates were excluded, 193 papers remained for review. Based on title and abstract review, irrelevant publications for those that did not satisfy our inclusion criteria were not considered, leaving 30 articles for full-text review. Following the full-text assessment of these articles, 13 papers [8–12, 15–22]remained for data extraction (Fig. 1).

From our risk of bias assessment of the included studies, we determined that 4 studies [10–12, 22] were associated with high risk of performance bias due to the authors explicitly stating that treating physicians were not blinded to their type of intervention [22] or that the trial was unblinded [10–12]. All other studies were at unclear risk of performance bias as not enough information was available for a conclusion to be made. Another 3 studies were prone to high risk of attrition bias due to insufficient details provided on missing data [17, 19, 21] (Fig. E1 and E2). Apart from these, we determined that the evidence provided by the included studies were still of robust quality (Table 1).

All studies were randomized controlled trials, reporting data on 6 trials, namely: the PARTNER 1 trial, PARTNER 2 trial, CoreValve US pivotal High Risk trial, the SURTAVI trial, Evolut Low Risk trial and NOTION trial. A mixed cohort of patients who were at low risk and high risk of surgery was included in our analysis. The TAVR valves compared were the CoreValve, Edwards SAPIEN, SAPIEN XT, Evolut R and Evolut Pro valves (Table E1).

All studies were multi-centre studies, with the majority taking place in the United States and Canada. Only the NOTION trial was carried out in Denmark and Sweden, and the Evolut Low Risk trial included centres based in Japan. A majority of the patients were over 70 years old. Apart from the study by Deeb et al. [16] reporting a significant difference between histories of diabetes mellitus in their TAVR and SAVR patient cohorts, the baseline characteristics of the TAVR and SAVR patients across the included studies were similar (Table E2).

### Meta-analysis of postoperative outcomes of TAVR and SAVR

All 13 studies were subjected to a meta-analysis, with the comparison between the postoperative primary and secondary outcomes of TAVR valves and SAVR valves.

### Incidence of paravalvular regurgitation

**Fig. 2:**
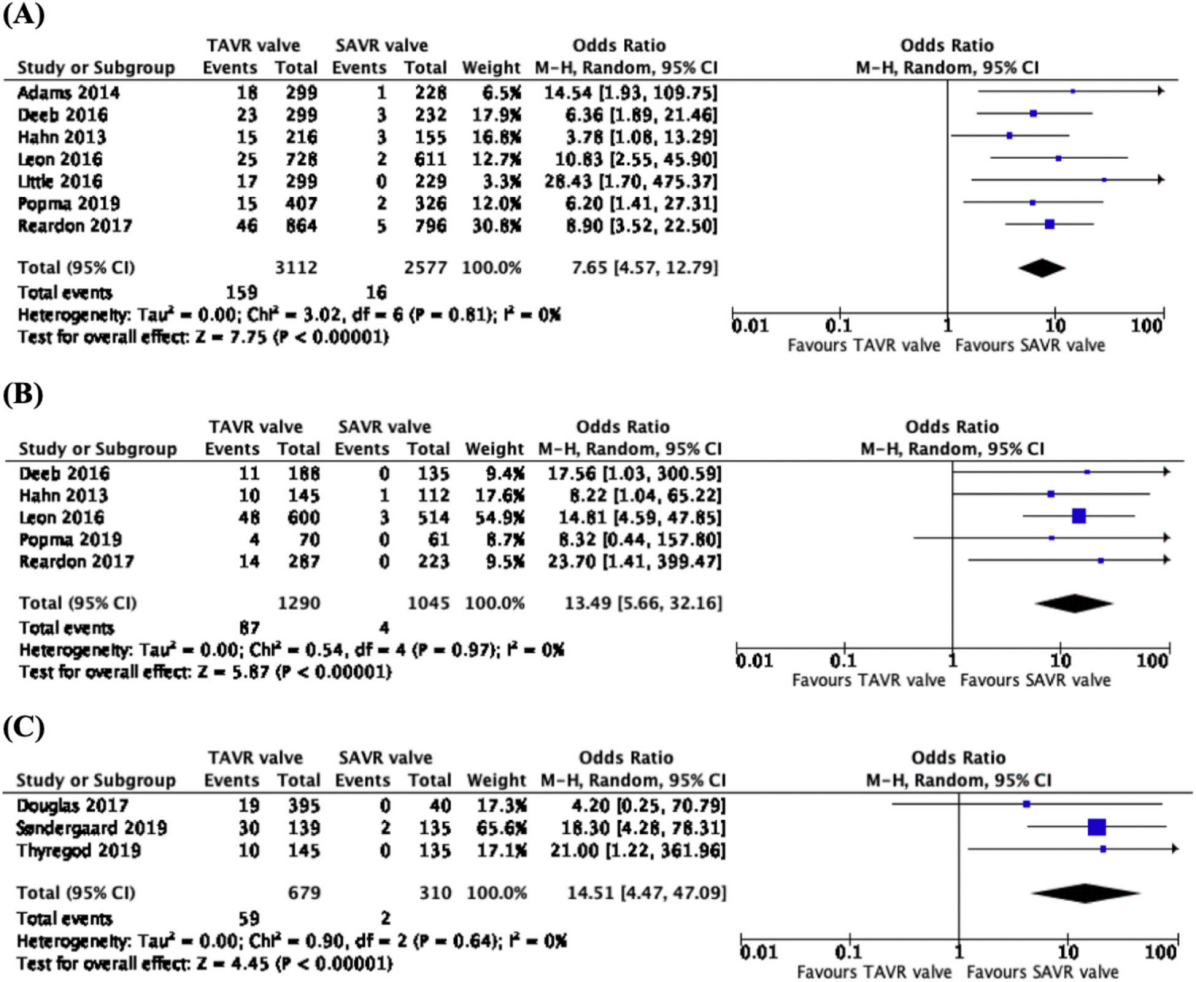
Forest plots of (**a**) 1-year (**b**) 2–3-year and (**c**) 5-year Paravalvular Regurgitation (Moderate or Severe). We observed higher rates of 1-year, 2–3-year and 5-year paravalvular regurgitation associated with the use of TAVR valves, as compared to SAVR valves

From the pooled analysis of 7 studies, 5689 patients, across 5 trials (CoreValve US Pivotal High Risk trial, PARTNER Cohort A trial, PARTNER 2 trial, Evolut Low Risk trial and SURTAVI trial), there was a significantly higher incidence of 1-year paravalvular regurgitation associated with the TAVR valve than the SAVR valve (OR: 7.65, CI: 4.57 to 12.79, *p* < 0.00001) (Fig. 2a). From the data of 5 studies, 2335 patients from 4 different trials (CoreValve US Pivotal High Risk trial, PARTNER Cohort A trial, Evolut Low Risk trial and PARTNER 2 trial), there was a significantly higher rate of 2–3-year paravalvular regurgitation in patients with TAVR valve than those with the SAVR valve (OR: 13.49, CI: 5.66 to 32.16, *p* < 0.00001) (Fig. 2b). Comparing 3 studies, with data reported on 989 patients from 2 trials (PARTNER trial and NOTION trial), there were more incidences of 5-year paravalvular regurgitation associated with the TAVR valve than the SAVR valve (OR: 14.51, CI: 4.47 to 47.09, *p* < 0.00001) (Fig. 2c).

### Incidence of moderate or severe aortic regurgitation

**Fig. 3:**
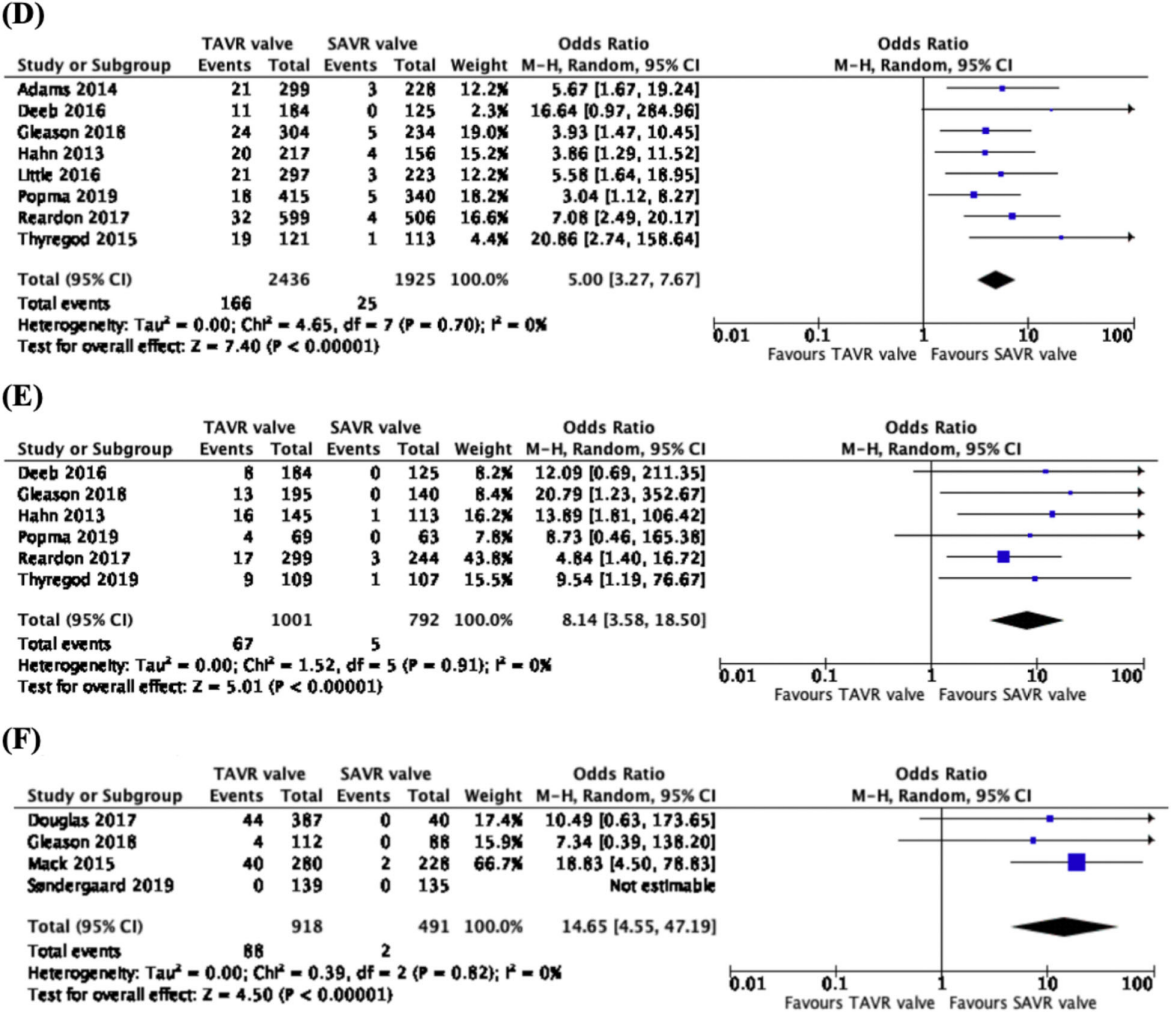
Forest plots of (**d**) 1-year, (**e**) 2–3-year and (**f**) 5-year Moderate or Severe Aortic Regurgitation. We observed higher rates of 1-year, 2–3-year and 5-year moderate or severe aortic regurgitation associated with the use of TAVR valves, as compared to SAVR valves

From 8 studies, 4361 patients and across 5 trials (CoreValve US Pivotal trial, CoreValve US High risk pivotal trial, PARTNER Cohort A, Evolut Low Risk trial and the NOTION trial), we observed a higher rate of 1-year moderate or severe aortic regurgitation in the TAVR valve cohort than the SAVR cohort (OR: 5.00, CI: 3.27 to 7.67, *p* < 0.00001) (Fig. 3d). From the pooled analysis of 6 studies, 1793 patients and 5 trials (CoreValve US Pivotal trial, CoreValve US High Risk trial, PARTNER Cohort A trial, Evolut Low Risk trial and NOTION trial), there was a higher rate of 2–3-year moderate or severe aortic regurgitation in the TAVR valve group as compared to the SAVR valve group (OR: 8.14, CI: 3.58 to 18.50, *p* < 0.00001) (Fig. 3e). Comparing the results of 4 studies and 1409 patients from 4 trials (PARTNER trial, CoreValve US High Risk trial, PARTNER 1 trial, NOTION trial), there was a significantly higher rate of 5-year moderate or severe aortic regurgitation in patients with the TAVR valve than those with the SAVR valve (OR: 14.65, CI: 4.55 to 47.19, *p* < 0.00001) (Fig. 3f).

### Reintervention rates

**Fig. 4:**
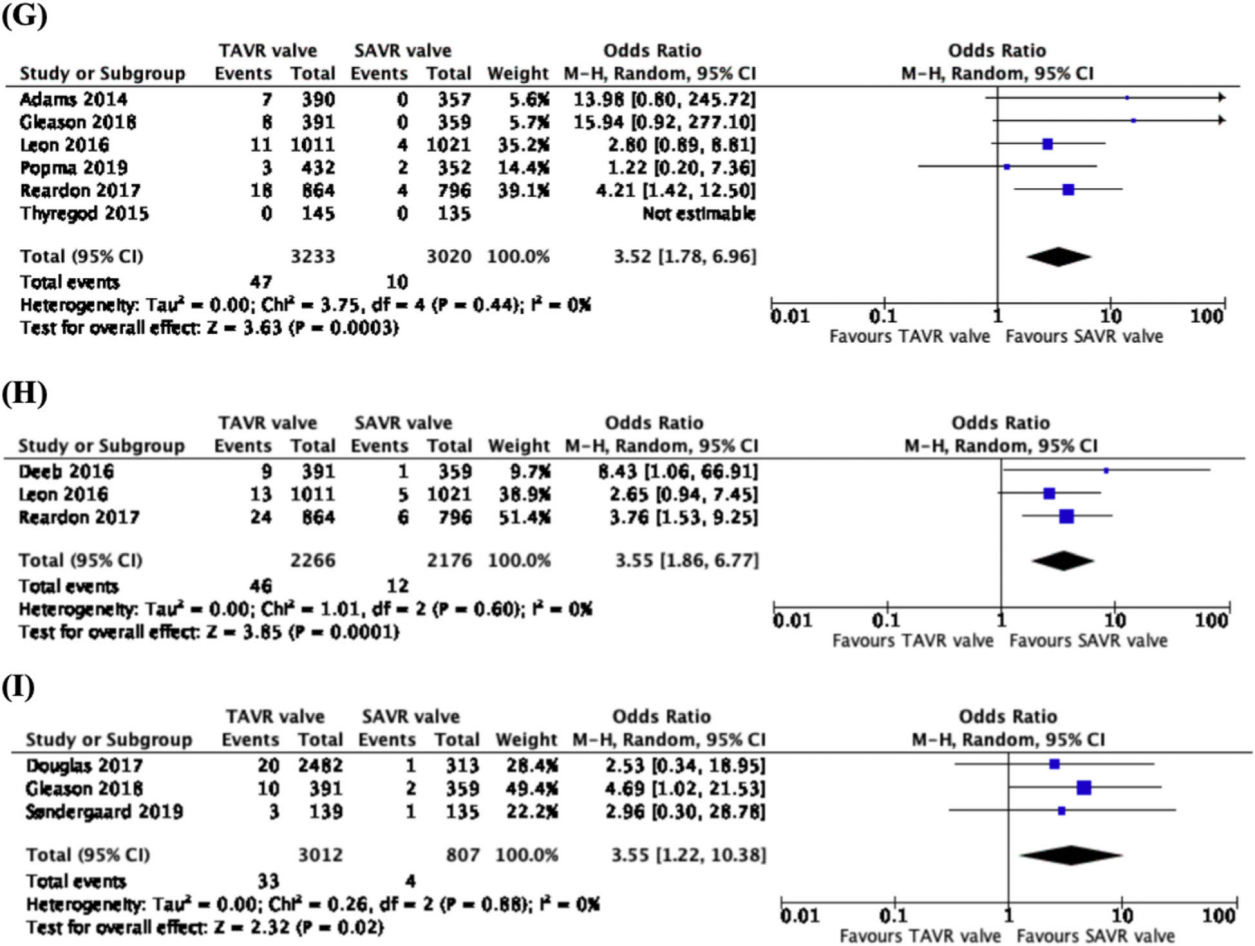
Forest plots of (**g**) 1-year, (**h**) 2–3-year and (**i**) 5-year Reintervention Rates. We observed higher rates of 1-year, 2–3-year and 5-year reintervention rates associated with the use of TAVR valves, as compared to SAVR valves

Comparing data from 6 studies, 6253 patients, across 5 trials (CoreValve US Pivotal High Risk trial, PARTNER 2 trial, SURTAVI trial, Evolut Low Risk trial and NOTION trial), there was a higher rate of 1-year reintervention associated with the TAVR valve, as compared to the SAVR valve (OR: 3.52, CI: 1.78 to 6.96, *p* = 0.0003) (Fig. 4g). With data from 3 studies and 4442 patients across 3 independent trials (CoreValve US Pivotal High Risk trial, PARTNER 2 trial and SURTAVI trial), there was a higher rate of 2–3-year reintervention reported with the TAVR valve than the SAVR valve (OR: 3.55, CI: 1.86 to 6.77, *p* = 0.0001) (Fig. 4h). From 3 studies, 3819 patients, across 3 trials, (PARTNER trial, CoreValve US Pivotal High Risk trial and NOTION trial), there was a significantly higher rate of 5-year reintervention rates observed with the TAVR valve as compared to the SAVR valve (OR: 3.55, CI: 1.22 to 10.38, *p* = 0.02) (Fig. 4i). One study (Thyregod et al. [11]) was excluded due to the data reported being the same as an already included study from the same trial.

### Incidence of endocarditis, all-cause mortality and mortality due to cardiovascular diseases

**Fig. 5:**
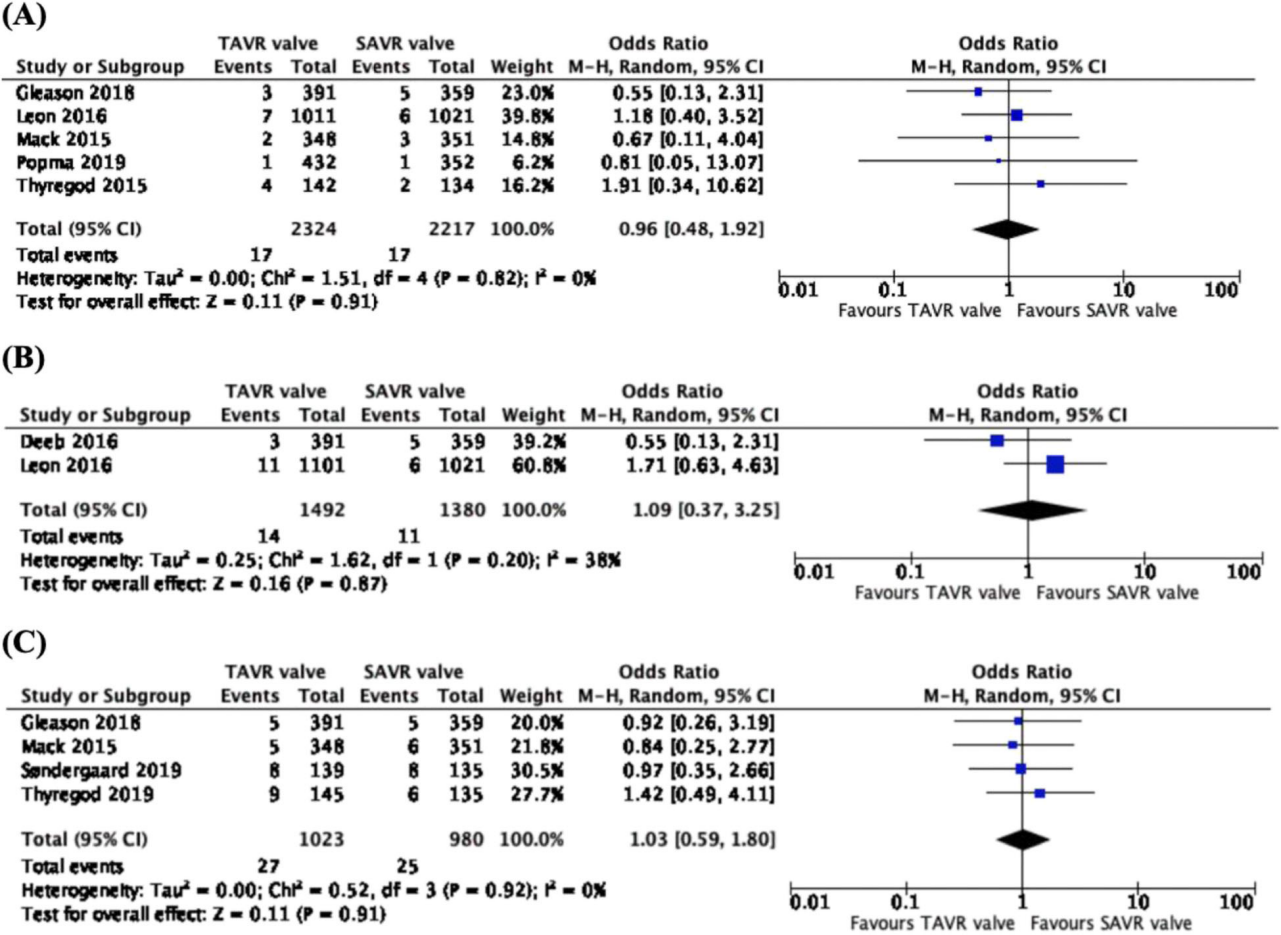
Forest plots of (**a**) 1-year, (**b**) 2–3-year and (**c**) 5-year Endocarditis. We observed no statistical difference in 1-year, 2–3-year and 5-year endocarditis between TAVR and SAVR valves

**Fig. 6:**
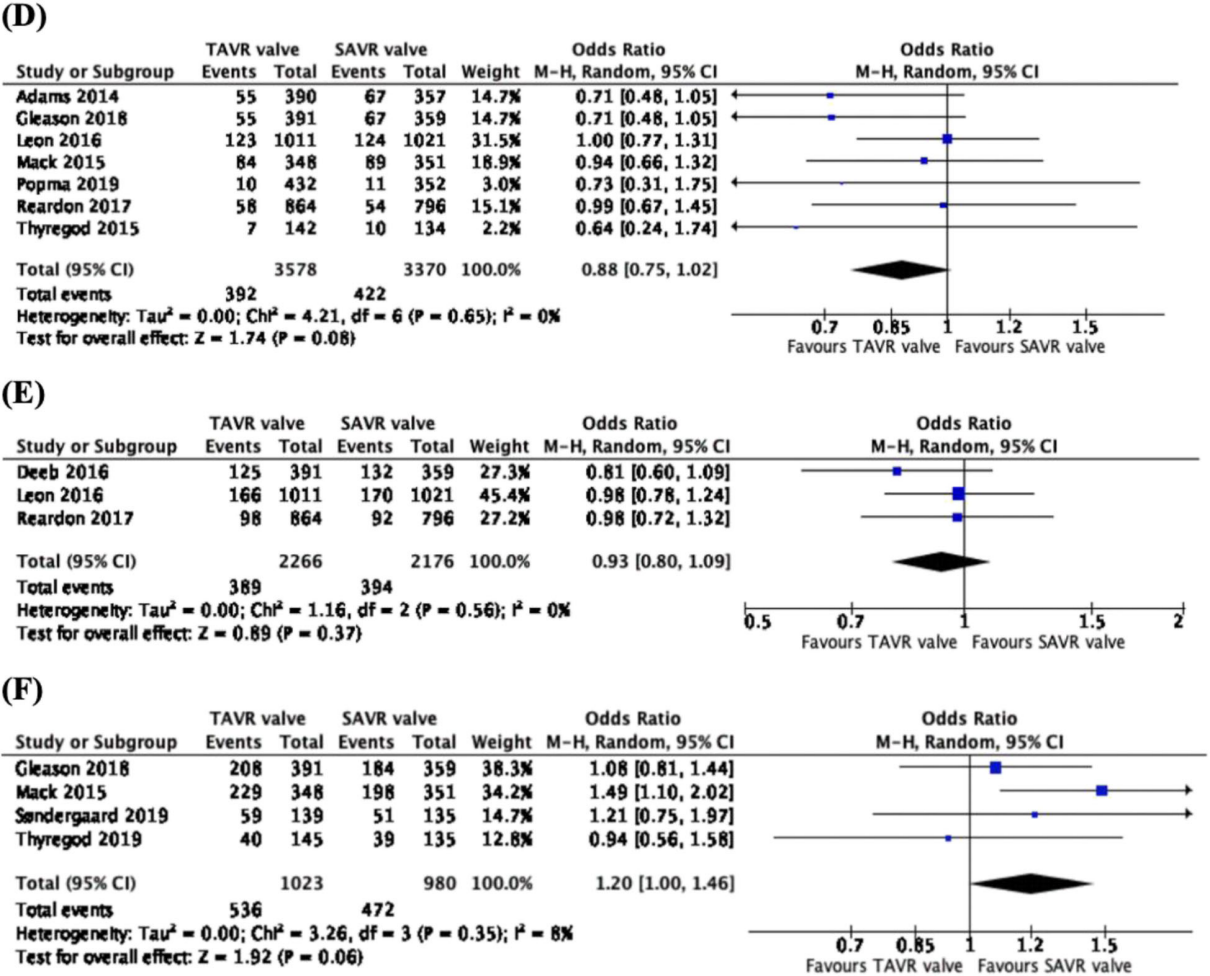
Forest plots of (**d**) 1-year, (**e**) 2–3-year and (**f**) 5-year All-cause Mortality. We observed no statistical difference in 1-year, 2–3-year and 5-year endocarditis between TAVR and SAVR valves

**Fig. 7:**
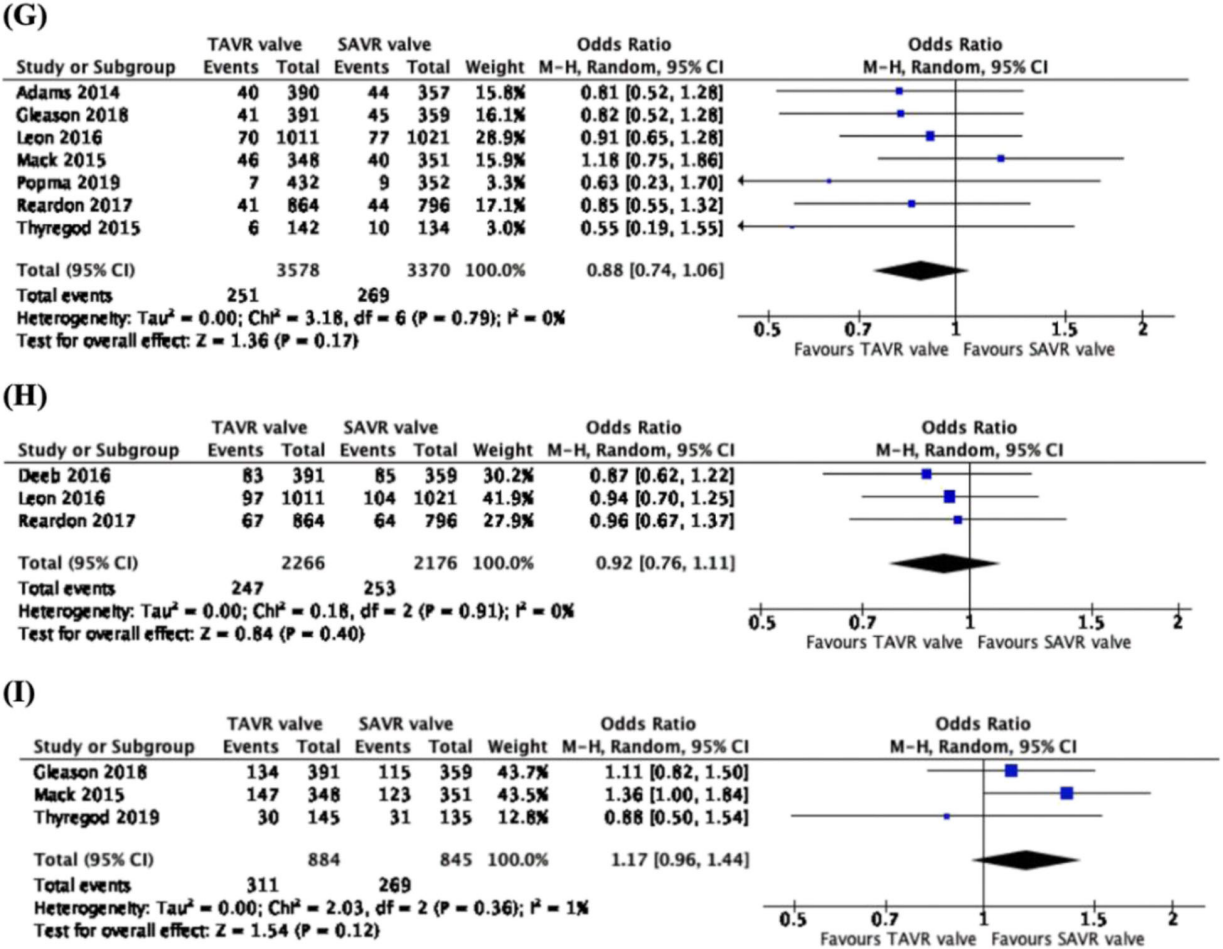
Forest plots of (**g**) 1-year, (**h**) 2–3-year and (**i**) 5-year Mortality Due to Cardiovascular Reasons. We observed no statistical difference in 1-year, 2–3-year and 5-year endocarditis between TAVR and SAVR valves

We observed no statistical differences in 1-year (OR: 0.96, CI: 0.48 to 1.92, *p* = 0.91), 2–3 year (OR: 1.09, CI: 0.37 to 3.25, *p* = 0.87) and 5-year (OR: 1.03, CI: 0.59 to 1.80, *p* = 0.91) incidence of endocarditis (Fig. 5), 1-year (OR: 0.88, CI: 0.75 to 1.02, *p* = 0.08), 2–3 year (OR: 0.93, CI: 0.80 to 1.09, *p* = 0.37) and 5-year (OR: 1.20, CI: 1.00 to 1.46, *p* = 0.06) all-cause mortality (Fig. 6) and 1-year (OR: 0.88, CI: 0.74 to 1.06, *p* = 0.17), 2–3 year (OR: 0.92, CI: 0.76 to 1.11, *p* = 0.40) and 5-year (OR: 1.17, CI: 0.96 to 1.44, *p* = 0.12) mortality due to cardiovascular diseases (Fig. 7).

## Discussion

According to the definition of structural valve deterioration (SVD) proposed by Dvir et al [23], SVD is composed of 4 stages (Stage 0 to 3), with stage 2 comprising of 3 ‘sub-stages’, namely Stages 2S (stenosis), 2R (moderate regurgitation) and 2RS (moderate regurgitation and stenosis). Following this definition, paravalvular regurgitation and infective endocarditis are not factors that directly lead to a diagnosis of SVD but may eventually contribute to the development of early stage SVD [23]. We observed no significant differences in the incidence of 1-year, 2–3 year or 5-year endocarditis between the TAVR valve and SAVR valve cohorts. However, our results of higher rates of 1-year, 2–3 year and 5-year paravalvular regurgitation may suggest a greater likelihood of TAVR valves developing SVD in the long run.

In the same definition, SVD Stage 2 concerns “morphological abnormalities of valve leaflets associated with haemodynamic dysfunction” [23]. The authors defined “haemodynamic dysfunction” as the incidence of either valvular stenosis or regurgitation, with moderate regurgitation graded SVD Stage 2R [23]. Finally, SVD Stage 3, the most severe stage, is defined by the presence of severe stenosis or regurgitation. At this stage, reintervention is recommended if the patient is symptomatic [23]. Therefore, our results of higher rates of moderate or severe aortic regurgitation associated with TAVR valves may also be indicative of a higher rate of SVD in the 1-year, 2–3 year and 5-year period as compared to SAVR valves, which would then suggest that early-generation TAVR valves might be less durable in the long term than SAVR valves overall. In prior research, studies have reported that more patients with TAVR developed higher rates of prosthesis regurgitation. Kodali et al [24] described that paravalvular regurgitation was more common after TAVR and Athappan et al [25] reported that moderate or severe aortic regurgitation was more frequent after TAVR as well. Our observations of higher rates of moderate or severe aortic regurgitation and paravalvular regurgitation were thus consistent with the available literature.

While only 4 studies reported their findings on SVD [10, 11, 18, 22], the authors of these studies utilised different definitions to determine a diagnosis of SVD and thus could not be compared. Instead, most studies reported reintervention rates, with some specifying the reason for reintervention being due to decreased haemodynamic performance or evidence of structural deterioration on echocardiographic examination of the valves [10, 11, 17]. Hence, the higher rates of reintervention in the 1-year, 2–3 year and 5-year period for the TAVR valves could also be taken as an indirect measure of SVD, and once again representative of TAVR valves having poorer structural durability as compared to SAVR valves.

On another note, with no significant differences observed in 1-year, 2–3 year or 5-year endocarditis, all-cause mortality and mortality due to cardiovascular reasons, it would seem that despite the increased susceptibility to SVD associated with TAVR valves, these might not lead to adverse complications in patients, and TAVR valves are likely to be safe for use in a mixed cohort of patients with aortic stenosis. However, it is also important to take into account that majority of the patients in the included studies were over 70 years old, which could have been a factor that contributed to this observation. We determined that a possible reason for the increased susceptibility to structural valve deterioration associated with the TAVR valves could be due to the fact that they are a relatively newer technology compared to their SAVR counterparts, and that more modifications to the designs of these early-generation valves could compensate for this discrepancy.

### Limitations

The main limitation of this study was its inability to compare values from a variety of echocardiographic variables due to a lack of reported data in literature, which made it difficult for the incidence of structural valve deterioration to be directly compared across all studies. Instead, we could only predict the susceptibility of these valves to SVD based on aortic regurgitation, paravalvular leak and reintervention rates. Additionally, echocardiographic values would have allowed for a more thorough analysis of the 1-year, 2–3-year and 5-year haemodynamic performance of the TAVR and SAVR valves. Another limitation we faced was the scarcity of randomised controlled trials available in literature comparing SAVR valves with newer TAVR valves, such as Medtronic Evolut R and Edwards SAPIEN 3 to name a few. This restricted the scope of our analysis to evaluating mainly the outcomes of early-generation TAVR valves compared to SAVR valves.

### Future prospective

In future, more randomised controlled trials reporting their findings on the 5-year structural durability of TAVR valves will be needed. More studies should also report data on echocardiographic variables in order to better assess the performance of the valves.

## Conclusion

TAVR valves may be associated with higher rates of 1-year, 2–3 year and 5-year moderate or severe aortic regurgitation, paravalvular leak and reintervention than SAVR valves. This could be indicative of TAVR valves being more susceptible to SVD and hence potentially less durable in the long term than SAVR valves.

## Supplementary information

**Additional file 1: Figure E1.** review authors’ judgements about each risk of bias item presented as percentages across all included studies. **Figure E2.** review authors’ judgements about each risk of bias item for each included study. **Table E1.** Summary of Included Studies.** Table E2.** Baseline Characteristics of Patients in the Included Studies.

## Data Availability

Not applicable.

## Abbreviations

TAVR: Transcatheter aortic valve replacement
SAVR: Surgical aortic valve replacement
PRISMA: Preferred reporting items for systematic reviews and meta-analyses for systematic review
PARTNER: Placement of AoRTic TraNscathetER Valve Trial
SURTAVI: Surgical Replacement and Transcatheter Aortic Valve Implantation
NOTION: Nordic Aortic Valve Intervention
Stage 2S: Stage 2 with stenosis
Stage 2R: Stage 2 with moderate regurgitation
Stage 2RS: Stage 2 with moderate regurgitation and stenosis
SVD: Structural Valve Deterioration
