# Supplementary material for: Structural durability of early-generation Transcatheter aortic valve replacement valves compared with surgical aortic valve replacement valves in heart valve surgery: a systematic review and meta-analysis
Source: J Cardiothorac Surg. 2020 Jun 8;15:127. doi: 10.1186/s13019-020-01170-7 (PMC7278207; doi:10.1186/s13019-020-01170-7)
Supplement: Supplementary file 1 — Additional file 1: Figure E1. review authors’ judgements about each risk of bias item presented as percentages across all included studies. Figure E2. review authors’ judgements about each risk of bias item for each included study. Table E1. Summary of Included Studies. Table E2. Baseline Characteristics of Patients in the Included Studies. [file 13019_2020_1170_MOESM1_ESM.docx]

**Figure E1: Risk of Bias Graph and Risk of Bias Summary**

**Figure E2: Risk of Bias Summary**

**Table E1: Summary of Included Studies**

| Study No. | First author, year | Study Type | Number of centres | Study period | Trial | Follow-up years | Number of patients | Valves used |
| --- | --- | --- | --- | --- | --- | --- | --- | --- |
| 1 | Adams et. al., 2014^10^ | Randomised Controlled Trial | 45 | February 2011 through September 2012 | CoreValve US Pivotal High Risk | 1 | 747 (390/357) | CoreValve |
| 2 | Deeb et. al., 2016^11^ | Randomised Controlled Trial | 45 | February 2011 to September 2012 | CoreValve US Pivotal High Risk | 3 | 750 (391/359) | CoreValve |
| 3 | Douglas et. al., 2017^12^ | Randomised Controlled Trial | 45 | May 11, 2007, through August 28, 2009 | PARTNER trial | 5 | 2795 (2482/313) | Edwards SAPIEN |
| 4 | Gleason et. al., 2018^13^ | Randomised Controlled Trial | 45 | February 2011 to September 2012 | CoreValve US Pivotal High Risk | 5 | 744 (390/354) | CoreValve |
| 5 | Hahn et. al., 2013^14^ | Randomised Controlled Trial | 45 | May 11, 2007, through August 28, 2009 | PARTNER Cohort A | 2 | 699 (348/351) | Edwards SAPIEN |
| 6 | Leon et. al., 2016^15^ | Randomised Controlled Trial | 57 | December 2011 tto November 2013 | PARTNER 2 | 2 | 2032 (1101/1021) | Balloon-expandable SAPIEN XT heart-valve system (Edwards Lifesciences) |
| 7 | Little et. al., 2016^16^ | Randomised Controlled Trial | 45 | February 2011 to September 2012 | CoreValve US Pivotal High Risk | 1 | 795 (389/353) | CoreValve |
| 8 | Mack et. al., 2015^17^ | Randomised Controlled Trial | 25 | May 11, 2007, through August 28, 2009 | PARTNER | 5 | 699 (348/351) | Balloon-expandable SAPIEN XT heart-valve system (Edwards Lifesciences) |
| 9 | Popma et. al., 2019^18^ | Randomised Controlled Trial | 86 | March 28, 2016, to November 27, 2018 | Evolut Low Risk | 2 | 1403 (725/678) | CoreValve, Evolut R, or Evolut PRO; Medtronic |
| 10 | Reardon et. al., 2017^19^ | Randomised Controlled Trial | 87 | June 19, 2012, to June 30, 2016 | SURTAVI | 2 | 1660 (864/796) | CoreValve and the Evolut-R bioprosthesis valves |
| 11 | Søndergaard et. al., 2019^20^ | Randomised Controlled Trial | 87 | December 2009 to April 2013 | NOTION | 6 | 274 (139/135) | Mosaic, epic, trifecta, perimount, sorin mitroflow (surgical valves) vs CoreValve |
| 12 | Thyregod et. al., 2019^21^ | Randomised Controlled Trial | 87 | December 2009 to April 2013 | NOTION | 5 | 280 (145/135) | Stented bioprosthetic aortic valve vs. CoreValve self-expanding bioprosthesis (Medtronic, Plc, Minneapolis, MN) |
| 13 | Thyregod et. al., 2015^22^ | Randomised Controlled Trial | 87 | December 2009 to April 2013 | NOTION | 1 | 280 (145/135) | stented bioprosthetic aortic valve vs. CoreValve self-expanding bioprosthesis (Medtronic, Plc, Minneapolis, MN) |

**Table E2 (continued): Baseline Characteristics of Patients in the Included Studies**

| First author, year | Age, y | | | Male, % | | | Body surface area, m^2^ | | | Hypertension, % | | | Peripheral Vascular Disease, % | | |
| --- | --- | --- | --- | --- | --- | --- | --- | --- | --- | --- | --- | --- | --- | --- | --- |
|  | TAVR | SAVR | p-value | TAVR | SAVR | p-value | TAVR | SAVR | p-value | TAVR | SAVR | p-value | TAVR | SAVR | P-value |
| Adams et. al., 2014^10^ | 83.1±7.1 | 83.2±6.4 | NA | 53.1 | 52.4 | NA | NA | NA | NA | 95.1 | 96.1 | NA | 41.1 | 41.7 | NA |
| Deeb et. al., 2016^11^ | 83.2±7.1 | 83.3 ± 6.4 | 0.82 | 52.9 | 52.4 | 0.88 | NA | NA | NA | 95.1 | 95.1 | 0.52 | 41.0 | 42.0 | 0.77 |
| Douglas et. al., 2017^12^ | 84.5 ± 7.15 | 84.5 (6.33) | NA | 52.5 | 57.2 | NA | NA | NA | NA | 92.0 | 93.9 | NA | 70.0 | 67.1 | NA |
| Gleason et. al., 2018^13^ | 83.2 | 83.3 | NA | NA | NA | NA | NA | NA | NA | NA | NA | NA | NA | NA | NA |
| Hahn et. al., 2013^14^ | 83.6±6.7 | 84.5±6.3 | 0.07 | 57.8 | 56.7 | 0.82 | 1.8±0.3 | 1.8±0.2 | 0.65 | NA | NA | NA | 43.2 | 41.6 | 0.7 |
| Leon et. al., 2016^15^ | 81.5±6.7 | 81.7±6.7 | NA | 54.2 | 54.8 | NA | NA | NA | NA | NA | NA | NA | 27.9 | 32.9 | NA |
| Little et. al., 2016^16^ | 83.2 ± 7.1 | 83.3 ± 6.4 | NA | 53.0 | 51.8 | NA | 1.8±0.2 | 1.9±0.2 | NA | NA | NA | NA | 40.9 | 41.0 | NA |
| Mack et. al., 2015^17^ | 83.6±6.8 | 84.5±6.4 | NA | 57.8 | 56.7 | NA | NA | NA | NA | NA | NA | NA | 43.2 | 41.6 | NA |
| Popma et. al., 2019^18^ | 74.1±5.8 | 73.6±5.9 | NA | 64.0 | 66.2 | NA | NA | NA | NA | 84.8 | 82.6 | NA | 7.5 | 8.3 | NA |
| Reardon et. al., 2017^19^ | 79 ±6.2 | 79.7 ± 6.1 | NA | 57.6 | 55.0 | NA | 1.9±0.2 | 1.9±0.2 | NA | 92.7 | 90.3 | NA | 30.8 | 29.9 | NA |
| Søndergaard et. al., 2019^20^ | 79.4±4.9 | 78.8±4.6 | 0.326 | 52.5 | 53.3 | 0.893 | NA | NA | NA | NA | NA | NA | 4.3 | 6.7 | 0.393 |
| Thyregod et. al., 2019^21^ | 79.2±4.9 | 79.0±4.7 | NA | 53.8 | 52.6 | NA | NA | NA | NA | 17.9 | 76.3 | NA | 4.1 | 6.7 | NA |
| Thyregod et. al., 2015^22^ | 79.2±4.9 | 79.0±4.7 | NA | 53.8 | 52.6 | NA | NA | NA | NA | 17.9 | 76.3 | NA | 4.1 | 6.7 | NA |

**Table E2 (continued): Baseline Characteristics of Patients in the Included Studies**

| First author, year | History of pulmonary disease, % | | | Coronary Artery Disease, % | | | Diabetes Mellitus, % | | | Prior CABG, % | | | Prior Atrial Fibrillation, % | | |
| --- | --- | --- | --- | --- | --- | --- | --- | --- | --- | --- | --- | --- | --- | --- | --- |
|  | TAVR | SAVR | p-value | TAVR | SAVR | p-value | TAVR | SAVR | p-value | TAVR | SAVR | p-value | TAVR | SAVR | P-value |
| Adams et. al., 2014^10^ | NA | NA | NA | 75.4 | 75.9 | NA | 34.9 | 45.4 | NA | 29.5 | 31.1 | NA | 40.9 | 45.9 | NA |
| Deeb et. al., 2016^11^ | NA | NA | NA | 75.4 | 76.0 | 0.85 | 34.8 | 45.1 | 0.004 | 29.4 | 31.5 | 0.54 | 41.0 | 46.0 | 0.17 |
| Douglas et. al., 2017^12^ | 62.6 | 67.4 | NA | 77.8 | 57.2 | NA | 36.9 | 40.9 | NA | NA | NA | NA | NA | 67.1 | NA |
| Gleason et. al., 2018^13^ | NA | NA | NA | NA | NA | NA | NA | NA | NA | NA | NA | NA | NA | NA | NA |
| Hahn et. al., 2013^14^ | 43.7 | 43.0 | 0.88 | 74.7 | 76.2 | 0.66 | NA | NA | NA | NA | NA | NA | 40.7 | 43.6 | 0.6 |
| Leon et. al., 2016^15^ | 31.8 | 30.0 | NA | 69.2 | 66.5 | NA | 37.7 | 34.2 | NA | 23.6 | 25.6 | NA | 31.0 | 35.2 | NA |
| Little et. al., 2016^16^ | 44.7 | 44.2 | NA | 75.3 | 75.6 | NA | NA | NA | NA | NA | NA | NA | 40.7 | 45.6 | NA |
| Mack et. al., 2015^17^ | 43.7 | 43.0 | NA | NA | NA | NA | NA | NA | NA | 42.5 | 43.6 | NA | 40.7 | 43.6 | NA |
| Popma et. al., 2019^18^ | 15.0 | 18.0 | NA | NA | NA | NA | 31.4 | 30.5 | NA | 2.5 | 2.1 | NA | 15.4 | 14.5 | NA |
| Reardon et. al., 2017^19^ | NA | NA | NA | 62.6 | 64.2 | NA | 34.1 | 34.8 | NA | 16.0 | 17.2 | NA | 28.1 | 26.5 | NA |
| Søndergaard et. al., 2019^20^ | 12.2 | 11.9 | 0.923 | NA | NA | 0.893 | 17.3 | 20.7 | 0.463 | NA | NA | NA | 29.0 | 24.8 | 0.393 |
| Thyregod et. al., 2019^21^ | 11.7 | 11.9 | NA | NA | NA | NA | 17.9 | 20.7 | NA | NA | NA | NA | 27.8 | 25.6 | NA |
| Thyregod et. al., 2015^22^ | 11.7 | 11.9 | NA | NA | NA | NA | 17.9 | 20.7 | NA | NA | NA | NA | 27.8 | 25.6 | NA |

CABG: Coronary Artery Bypass Graft surgery

**Table E2 (continued): Baseline Characteristics of Patients in the Included Studies**

| First author, year | Pre-existing pacemaker, % | | | Prior Myocardial Infarction, % | | | STS score | | | NYHA functional class III or IV, % | | | Prior balloon valvuloplasty, % | | | |
| --- | --- | --- | --- | --- | --- | --- | --- | --- | --- | --- | --- | --- | --- | --- | --- | --- |
|  | TAVR | SAVR | p-value | TAVR | SAVR | p-value | TAVR | SAVR | p-value | TAVR | SAVR | p-value | TAVR | SAVR | P-value |  |
| Adams et. al., 2014^10^ | 23.3 | 21.3 | NA | 25.4 | 25.2 | NA | 7.3±3.0 | 7.5±3.4 | NA | 85.7 | 86.9 | NA | NA | NA | NA |  |
| Deeb et. al., 2016^11^ | 23.3 | 21.2 | 0.49 | NA | NA | NA | NA | NA | NA | NA | NA | NA | NA | NA | NA |  |
| Douglas et. al., 2017^12^ | NA | NA | NA | 25.9 | 29.0 | NA | NA | NA | NA | 95.0 | 94.9 | NA | NA | NA | NA |  |
| Gleason et. al., 2018^13^ | NA | NA | NA | NA | NA | NA | NA | NA | NA | NA | NA | NA | NA | NA | NA |  |
| Hahn et. al., 2013^14^ | NA | NA | NA | 26.5 | 29.8 | 0.66 | 11.8±3.2 | 11.7±3.4 | 0.61 | 94.3 | 94.0 | 0.89 | 13.2 | 10.0 | 0.2 |  |
| Leon et. al., 2016^15^ | 11.7 | 12.0 | NA | NA | NA | NA | NA | NA | NA | NA | NA | NA | 5.0 | 4.9 | NA |  |
| Little et. al., 2016^16^ | NA | NA | NA | 25.4 | 25.5 | NA | 7.3±3.0 | 7.5±3.4 | NA | 85.6 | 87.0 | NA | 6.2 | 5.9 | NA |  |
| Mack et. al., 2015^17^ | 19.8 | 21.8 | NA | NA | NA | NA | 11.8±3.3 | 11.7±3.5 | NA | 94.3 | 94.0 | NA | NA | NA | NA |  |
| Popma et. al., 2019^18^ | 3.2 | 3.8 | NA | 6.6 | 4.9 | NA | NA | NA | NA | 25.1 | 28.4 | NA | NA | NA | NA |  |
| Reardon et. al., 2017^19^ | 9.7 | 9.0 | NA | 14.5 | 13.9 | NA | 4.4±1.5 | 4.5±1.6 | NA | 60.2 | 58.2 | NA | NA | NA | NA |  |
| Søndergaard et. al., 2019^20^ | 3.6 | 4.4 | 0.72 | NA | NA | NA | 3.0±1.7 | 3.0±1.6 | 0.882 | 46.4 | 46.3 | 0.986 | NA | NA | NA |  |
| Thyregod et. al., 2019^21^ | 3.4 | 4.4 | NA | 5.5 | 4.4 | NA | 2.9±1.6 | 3.1±1.7 | NA | 48.6 | 45.5 | NA | NA | NA | NA |  |
| Thyregod et. al., 2015^22^ | 3.4 | 4.4 | NA | 5.5 | 4.4 | NA | 2.9±1.6 | 3.1±1.7 | NA | 48.6 | 45.5 | NA | NA | NA | NA |  |

STS: Society of Thoracic Surgery, NHYA: New York Heart Association
